# Thrombosis and Thrombotic Risk in Athletes

**DOI:** 10.3390/jcm13164881

**Published:** 2024-08-19

**Authors:** Ciro Miele, Cristina Mennitti, Alessandro Gentile, Iolanda Veneruso, Carmela Scarano, Aniello Vastola, Ilaria La Monica, Fabiana Uomo, Fernanda Iafusco, Filomena Capasso, Raffaela Pero, Valeria D’Argenio, Barbara Lombardo, Nadia Tinto, Pierpaolo Di Micco, Olga Scudiero, Giulia Frisso, Cristina Mazzaccara

**Affiliations:** 1 Department of Molecular Medicine and Medical Biotechnologies, Federico II University, Via Sergio Pansini 5, 80131 Napoli, Italy; ciro.miele1987@gmail.com (C.M.); cristinamennitti@libero.it (C.M.); alexgenti98@libero.it (A.G.); venerusoi@ceinge.unina.it (I.V.); scaranoc@ceinge.unina.it (C.S.); vastola92@gmail.com (A.V.); fabiana.uomo@gmail.com (F.U.); pero@unina.it (R.P.); barbara.lombardo@unina.it (B.L.); nadia.tinto@unina.it (N.T.); gfrisso@unina.it (G.F.); cristina.mazzaccara@unina.it (C.M.); 2 UOC Laboratory Medicine, Haematology and Laboratory Haemostasis and Special Investigations, AOU Federico II University of Naples, 80131 Naples, Italy; menacapasso@libero.it; 3 CEINGE-Biotecnologie Avanzate Franco Salvatore, Via G. Salvatore 486, 80145 Napoli, Italy; lamonica@ceinge.unina.it (I.L.M.); iafusco@ceinge.unina.it (F.I.); dargenio@ceinge.unina.it (V.D.); 4 Task Force on Microbiome Studies, University of Naples Federico II, 80100 Naples, Italy; 5 Department of Human Sciences and Quality of Life Promotion, San Raffaele Open University, 00100 Rome, Italy; 6 AFO Medicina, P.O. Santa Maria delle Grazie, Pozzuoli, ASL Napoli2 nord, 80076 Naples, Italy

**Keywords:** thrombosis, physical activity, anticoagulant therapy, doping, coagulation, microbiota

## Abstract

The hemostatic system is characterized by a delicate balance between pro- and anticoagulant forces, and the smallest alteration can cause serious events such as hemorrhages or thrombosis. Although exercise has been shown to play a protective role in athletes, several factors may increase the risk of developing venous thromboembolism (VTE), including hemoconcentration induced by exertion, immobilization following sports injuries, frequent long-distance flights, dehydration, and the use of oral contraceptives in female athletes. Biomarkers such as D-dimer, Factor VIII, thrombin generation, inflammatory cytokines, and leukocyte count are involved in the diagnosis of deep vein thrombosis (DVT), although their interpretation is complex and may indicate the presence of other conditions such as infections, inflammation, and heart disease. Therefore, the identification of biomarkers with high sensitivity and specificity is needed for the screening and early diagnosis of thromboembolism. Recent evidence about the correlation between the intensity of physical activity and VTE is divergent, whereas the repeated gestures in sports such as baseball, hockey, volleyball, swimming, wrestling, or, on the other hand, soccer players, runners, and martial art training represent a risk factor predisposing to the onset of upper and lower DVT. Anticoagulant therapy is the gold standard, reducing the risk of serious complications such as pulmonary embolism. The aim of this review is to provide a general overview about the interplay between physical exercise and the risk of thromboembolism in athletes, focusing on the main causes of thrombosis in professional athletes and underlying the need to identify new markers and therapies that can represent a valid tool for safeguarding the athlete’s health.

## 1. Introduction

Physical activity plays an important role in reducing the onset of numerous chronic diseases [1], such as cardiovascular disease [2,3,4], hypertension [5], diabetes [6], osteoporosis [7], and obesity [8]. On the other hand, exercise has been shown to affect the balance of the hemostatic system, promoting the formation of thrombi through a temporary increase in blood coagulation, platelet aggregation, and fibrinolytic activity. Generally speaking, venous thromboembolism (VTE), which encompasses deep vein thrombosis (DVT) and pulmonary embolism (PE), represents one of the main causes of vascular damage worldwide, right after acute myocardial infraction and stroke, with the number of cases estimated to be around 10 million every year [9]. Nevertheless, the pathophysiological mechanisms underlying the disease are still not fully understood [10]. Thrombi, consisting of a laminar structure formed by platelets, red blood cells, leukocytes, and fibrin, can occur in virtually all possible venous districts, commonly found in valve pockets and dilated sinuses of the lower limbs [11]. Physiologically, the hemostatic system is a balance between pro- and anticoagulant forces; shifting that prompts a hypercoagulable state may develop into a clinical manifestation, leading to a thromboembolic disease. This is the result of a complex mechanism that embroils not only factors involved in the coagulation cascade, but also their dynamic interactions with blood vessels, endothelial cells, platelets, and other cells in the circulation. Rudolf Virchow in 1856 suggested the involvement of three key elements in the triggering of a thrombotic event: stasis, endothelial damage, and hypercoagulability. Hence, causes of VTE are multifactorial and the clinical outcome is due to the synergy of single or multiple genetic, epigenetic, and/or acquired predisposing factors, exposing those affected to an improper clot formation [10,12]. Inherited risk factors for VTE encompass deficiencies of the natural inhibition of coagulation (Antithrombin, Protein C and Protein S) [13], genetic dysfibrinogenemia [14], or factor V Leiden [15] and prothrombin G20210A mutations [16]. On the other hand, acquired risk factors for VTE can trigger a hypercoagulable state following the increase in procoagulant factors, reduction in anticoagulant proteins, and activation of pro-inflammatory/autoimmune systems [17]. Major acquired conditions predisposing to thrombotic events include antiphospholipid syndrome [18], hyperomocisteinaemia [19], the presence of specific medical conditions (e.g., hematological diseases and cancer) [20], or drugs (chemotherapy or oral contraceptive therapy) [21,22]. Smoking, obesity, age, and pregnancy may further contribute to the disease [23]. In case of a co-existence of acquired and genetic factors predisposing to VTE, a low risk factor may have greater resonance if bundled together with a genetic factor predisposing to thrombosis, even at a young age. However, elderly patients (>60 years) with one or two acquired risk factors can potentially develop a thrombotic event even if they do not have genetic risk factor that predisposes them to thrombosis [16]. While athletes are commonly considered to have a lower risk of developing VTE compared to the general population, they are not completely immune to thrombotic events. Several cases of VTE have been reported in the literature, due to athletes being frequently exposed to a number of acquired sport-related hypercoagulability conditions, ranging from hemoconcentration induced by exertion, immobilization following sports injuries, frequent long-distance flights, dehydration, and the use of oral contraceptives in female athletes, all causing an increased blood clot tendency [24]. DVT is particularly common in weightlifters and baseball players, exposing these athletes to upper extremity DVT. The age range was between 14 and 29 years, and males are more affected than women [25]. At the same way, athletes who practice sport such as running and marathons could develop lower extremity DTV and, as previously reported, with a higher prevalence of male athletes affected.

Although VTE represents a significant health concern regardless of the occupation, it can have profound implications on the athletes’ professional careers, prompting important changes in their professional trajectories, either by prematurely ending their careers or causing them to face extended absences from games or training due to the necessity of long-term anticoagulant treatment [26]. A retrospective review of injury reports conducted by Bishop et al. [26], considering an all-male healthy athlete cohort playing within four major professional athletic leagues in the United States (National Hockey League, Major League Baseball, the National Basketball Association and the National Football League), highlighted how, whilst VTE remains a rare condition in sportspeople, athletes can still be exposed to a variety of acquired risk factors inducing thrombotic episodes and affecting their return to professional games, with a large number of athletes returning to the field only after an anticoagulant regimen or surgery. While it is not common for younger and healthier athletes to develop DVT or PE, they are still susceptible, especially when they possess multiple risk factors. A study conducted by Humme deals with two case reports concerning the onset of a thrombotic event in two female athletes, a 20-year-old collegiate female soccer athlete and 19-year-old collegiate female track athlete. Despite their ages, both athletes experienced DVT events after lower extremity trauma [27].

It is crucial for athletic trainers to be vigilant about recognizing the signs and symptoms of VTE and to encourage athletes to promptly report any concerning changes in their well-being. Female athletes should be encouraged to disclose their contraceptive methods and any alterations in usage throughout their athletic career. Those at a higher risk of developing DVT/PE should consider non-hormonal birth control options if feasible or consult a healthcare provider to determine the most suitable hormonal choices. It is essential for any medical professional closely involved with a team or working with athletes to be well versed in both the signs and symptoms of VTE and the predisposing factors that these athletes may possess.

In this scenario, our review aims to provide a general overview about the connection between physical exercise and thromboembolic risk in athletes, highlighting the main causes of thrombosis in professional athletes and the need to identify new markers and valid therapies for safeguarding the athlete’s health.

### Deep Vein Thrombosis

Three different mechanisms are involved in the onset of deep venous thrombosis (DVT), venous stasis, hypercoagulability, and endothelial dysfunction, also known as Virchow’s triad [28], that, alone or combination, could affect the athlete, and are related to the intensity and duration of exercise. Athletes are also exposed to the risk of venous thrombosis due to other factors such as trauma, immobilization, and long-term travel.

Although exercise could be a protective weapon against thrombosis with the correct balance between exercise-activated coagulation and the fibrinolytic system [29], recent studies have demonstrated a negative correlation between physical activity and thrombotic events.

Strenuous exercise or trauma could cause a rare condition known as upper extremity DVT (UEDVT), often called Paget–Schroetter’s syndrome or effort thrombosis, that mostly affects young athletes, with a higher prevalence in males than in females.

In most cases, effort thrombosis is at the level of axillary and subclavian veins, and it is due to recent trauma or strenuous and repetitive exercise involving the affected arm.

Effort thrombosis has been described in athletes involved in a wide variety of sports, including baseball, softball, hockey, volleyball, swimming, wrestling, martial arts, back packing, and billiards [28]. Symptoms include unilateral edema of the arm, a sensation of “heaviness”, intense arm pain, cyanosis, or even pulmonary embolism symptoms: dyspnea, chest pain radiating to the shoulder, palpitations, cough (with or without blood expectoration), asthenia, dizziness, and fever [30,31]. A possibly lethal complication is pulmonary embolism, which may occur in 36% the patients.

On the other hand, lower extremity DVT (LEDVT) has been described more frequently in athletes such as soccer players, runners, and martial artists [32]. This event appears post-trauma mostly at the level of the popliteal vein, posterior tibial vein, and peroneal vein. Clinical symptoms are characterized by unilateral edema of the lower limb, particularly present on the calf and ankle, tension or pain in the calf, moderate fever, and a positive Homans sign (calf pain on leg dorsiflexion) [33]. Pulmonary embolism and post-thrombotic syndrome (PTS) are two potential complications; pulmonary embolism may occur in 50% of untreated DVT patients and may cause death in 2.1% of cases. For healthy women, the use of combined oral contraceptives (COCs) may significantly increase the risk of venous thrombosis. A study by Moreira Sales et al. reported a case of a 21-year-old professional woman soccer player who developed deep vein thrombosis (DVT) followed by pulmonary embolism (PE). The case was associated with the use of combined oral contraceptives. She was treated with rivaroxaban for 5 months, with complete resolution of symptoms [34]. Another case report is about a male soccer player who suffered lower extremity trauma during a match. The development of DVT was a consequence of the trauma and was particularly significant because DVT related to sports injuries is rare and often underdiagnosed [35]. Both cases highlight the need for timely diagnosis and appropriate treatment to prevent severe complications in athletes who develop DVT and PE.

## 2. Pulmonary Embolism

Pulmonary embolism (PE) is a serious disease with catastrophic implications if not treated promptly. Together with deep vein thrombosis it constitutes what is defined as venous thromboembolism. The annual rate of PE in the population is around 1 case per 1000 people, but this value rises if age is considered (1.4 per 1000 people aged 40–49 to 11.3 per 1000 aged 80 years) [36]. The pathophysiological mechanism of PE in relation to sports involves the formation of a blood clot in an artery in the lung, which obstructs the flow of blood to the lung itself. In most cases, the thrombus originates in the deep venous system of the lower extremities; in rare cases, it may also derive from pelvic, renal, upper extremity veins, or the right heart chambers. Once the thrombus reaches the pulmonary bifurcation, it can lodge itself in the arteries and cause hemodynamic compromise. Several factors can contribute to the development of PE in athletes: prolonged periods of immobility during travel, recovery from injuries, or sedentary activities can lead to blood stasis and an increased risk of clot formation [37,38]; endothelial damage caused by high-intensity physical exertion and repetitive trauma associated with certain sports can cause damage to the lining of blood vessels (endothelium), triggering the activation of blood clotting factors and increasing the likelihood of clot formation; dehydration induced by inadequate fluid intake can lead to a hypercoagulable state, where the blood becomes more viscous and prone to clotting; or underlying genetic conditions predisposing to increase propensity for clotting formation, such as abnormalities in clotting factors or deficiencies in natural anticoagulant proteins. The clinical suspicion of PE in sports can be complicated by the fact that respiratory distress, chest pain, irregular heartbeat, and coughing are all very common symptoms among competing athletes, thus reinforcing the possibility of a misdiagnosis. Over the years, experience in the management, diagnosis, and use of risk assessments of this disorder has evolved, allowing the implementation of diagnostic and therapeutic systems. A correct differential diagnosis is necessary in pulmonary embolism, as often the symptoms can mimic those of other pathological conditions; in this regard, clinical scores in association with D-dimer testing strengthen the use and interpretation of diagnostic imaging. From a pathophysiological point of view, the presence of PE can cause serious alterations that lead to hemodynamic compromise, especially when the size of the clot is very large. The right ventricle (RV), in particular, is the one most affected by PE. Physiologically, it is equipped with a thin-walled structure which pumps against the low-pressure and low-resistance pulmonary circulation [39]. Oxygen-sensing mechanisms underlie pulmonary vascular resistance systems; therefore, in the context of a PE the RV afterload can be enhanced by two pathways: one due to mechanical obstruction, the other by hypoxic vasoconstriction [33]. Inflammatory mediators, such as thromboxane A2 and histamine, generated as a result of clot formation, further increase vasoconstriction. RV failure, however, occurs not only with increased vascular resistance, but with the reduction in contractility as well, due to RV myocardial ischemia and the increase in RV preload [39]. All these impairments of the RV can cause a dilation of the RV that triggers an interventricular septum flattening into the left ventricle (LV), reduction in LV filling, and obstructive shock [39]. In a 2016 study, Casals et al. noticed that over the course of a five-year-period examination, a minimum of 15 cases of PE were documented among basketball players, suggesting a potential higher incidence of PE in professional basketball players in the NBA and ACB leagues compared to individuals of the same age and gender in the general population. They concluded that there are compelling indications that basketball players may represent a particularly vulnerable group prone to developing PE [40]. 

### Thoracic Outlet Syndrome in Athletes

Thoracic outlet syndrome (TOS) encompasses a string of disorders involving nerve and blood compression in the thoracic outlet area of the vascular or neurologic bundle. Described for the first time by Peet et al. in 1956, it can be distinguished in a congenital, acquired, or traumatic form, the latter being the most common of the three forms. Athletes are the most affected, particularly in sports involving upper arm solicitation, but it can be frequently overlooked in this population due to the rarity of neurovascular compressive disease and since muscle hypertrophy is renowned for prompting vascular or neurogenic compression [41]. The anatomical region of the thoracic outlet encompass three sites that can be exposed to the compression: the interscalene triangle bordered by the anterior scalene muscle, the first rib at the bottom, and the middle scalene muscle; the costoclavicular space that encompass the brachial plexus and the subclavian vessels, delimited posteroinferiorly by the first rib and anteriorly by the subclavian muscle; and the inferior aspect of the clavicle, the sub-coracoid space. Three forms of TOS are distinguished: neurological, venous, and arterial. Neurological TOS, or N-TOS, is the most common form (90–95% of all TOS cases reported) and it is responsible for the compression and the following irritation of the brachial plexus nerves as they cross the scalene triangle at neck’s base. The pathophysiological mechanism involves a continuous cycle of repetitive injury, inducing fibrosis and hypertrophy of the scalene or pectoralis minor muscles. This is followed by the deposition of scar tissue onto the brachial plexus nerves themselves. Venus TOS (V-TOS) affects around 5%–10% of all TOS cases and involves thrombotic events on the upper arm veins and eventually leads to pulmonary embolism (Paget–Schroetter syndrome). This chronic condition is induced by repetitive venous damage following arm exercise, despite normal anatomical structures in the costoclavicular space. The latter encompasses the region between the clavicle, first rib, anterior scalene muscle, and the subclavius muscle. Over time, this condition evolves into a cycle of injury and tissue healing, accompanied by the gradual buildup of constricting scar tissue around the veins. Initially, individuals may not experience any symptoms for several years as the body compensates by developing alternate drainage pathways. However, when an acute blood clot forms and propagates within the primary subclavian vein, it obstructs both the main venous drainage and collateral circulation. This sudden blockage, occurring on top of the chronic obstruction, leads to rapid and spontaneous swelling of the entire arm, accompanied by cyanosis, heaviness, early fatigue, and pain during use. In some cases, this condition may also result in pulmonary embolism. The last form is the arterial TOS, or A-TOS, which leads to the restriction of blood flow or complete blockage of the axillary or subclavian artery, inducing upper limb ischemia. The diagnosis of TOS can be tricky and is made excluding other pathologies, due to the great heterogeneity of the symptoms (especially regarding N-TOS) and the lack of defined diagnostic criteria. Arterial thoracic outlet syndrome (TOS) is caused by the presence of subclavian artery pathology within the scalene triangle, which occurs due to underlying anatomical factors, typically involving skeletal abnormalities like a cervical rib, underdeveloped first rib, or clavicle fracture. It represents the least common type of TOS, comprising approximately 1–3% of patients in major clinical studies. Subclavian artery aneurysms develop as a consequence of post-stenotic dilation resulting from prolonged and consistent compression of the subclavian artery. This is predominantly observed in the context of skeletal irregularities, like a congenital cervical rib or a hypoplastic first rib. The post-stenotic dilation of the artery induces degeneration of the aneurysmal wall, leading to the formation of ulcers and thrombi that are prone to embolization towards distal regions. Even though there have been improvements regarding the knowledge of this syndrome, there are still many steps to take to deepen our knowledge about TOS, especially its neurological form. This is due primarily to the absence of defined diagnostic criteria, with limited specificity and a wide range of symptoms [42]. All of this is further exacerbated by limited experience owing to the rarity of TOS cases, making its recognition challenging [43]. Consequently, because of its heterogeneous manifestations there is the need to conduct dedicated investigations for each distinct scenario to improve differential diagnosis, particularly among the athlete’ population who are highly vulnerable to TOS.

Preventative measures are key to reducing the risk of developing TOS, especially for athletes who perform activities with repetitive arm movements or poor posture. First of all, good posture is one of the most important ways to prevent TOS. Keeping your shoulders back and your head aligned over your spine reduces strain on your thoracic output. Moreover, practicing strengthening exercises that target the muscles of the shoulders, chest, and upper back can help stabilize the shoulder blades and reduce compression on the thoracic outlet. Likewise, regular stretching of the chest muscles can prevent tightness that could contribute to TOS. Athletes who practice repetitive movements should ensure they take regular breaks and vary their activities to prevent repetitive strain on the shoulders and neck [35].

By implementing these preventative measures, athletes could significantly reduce their risk of developing TOS, thereby avoiding the discomfort and potential complications associated with this condition.

## 3. Thrombophilia and Sport Events

Several cases of athletes with thrombotic events have been reported in the literature, showing a higher prevalence of DVT following intense training, competition travels, and after immobilization due to injuries [35,44,45]. Conversely, few cases of athletes with DVT have been reported as a consequence of mild to moderate sporting practice [44].

### 3.1. Acquired Thrombophilia and Hypophibrinolysis in Athletes

Although physical activity has pleiotropic beneficial effects, preventing the development of acute myocardial infarction and stroke, the existence of a risk–benefit paradox between blood coagulation and sport is broadly known. Strenuous physical activity may result in the imbalance of the hemostatic system generated by transient triggering of blood coagulation, platelet activation, and increased fibrinolytic activity. It is estimated that 1 in 1.000 athletes may suffer from a post-exercise thromboembolic event, similar to data obtained from the general non-athlete population. Long-haul travels, hemoconcentration induced by dehydration, and trauma are a few of the acquired conditions reported that can be magnified when combined with an inheritable clotting disorder already present in some athletes. DVT symptoms in sportspeople are often underestimated and not easily recognized, since they can be confused with pains induced by intense workouts or injuries. The acquired prothrombotic environment that is established in sportspeople follows the Virchow’s triad, encompassing hypercoagulability, endothelial damage, and stasis. Increased venous blood flow can lead to high shear stress forces directly affecting the vascular walls, which in response will express more tissue factor (TF) on their endothelial membrane, whose function is to trigger blood clotting. The hemostatic balance can be further impaired if water intake is reduced during intense physical exercise; dehydration, in fact, provokes an increase in blood viscosity due to a relative rise in erythrocyte count and a reduction in plasma volume. Additional elements such as high-altitude training and performance-enhancing supplements develop a hypercoagulable environment, caused by an increase in erythropoiesis and blood viscosity, changes in the molecular markers of the coagulation/fibrinolysis system, and enhancement in platelet activation and cholesteremia. The cardiovascular system in elite athletes undergoes a series of substantial adaptations as a consequence of intense training [46]. Different circumstances can result in a thrombotic event, such as bradycardia, overdevelopment of musculature, and prolonged periods of immobility. Cardiac molecular and cellular reshaping prompted by excessive training may elevate the risk of VTE, creating a greater propensity for blood pooling and venous stasis [31,47,48]. The latter can also be developed in many athletes due to muscular hypertrophy, leading to the compression of the venous system and promoting venous stasis and increasing VTE risk [47,49]. Athletes regularly have to endure long-haul travels, in order to participate in sport tournaments or training, exposing them to an increased thrombosis risk. This can be caused by prolonged periods of immobility that lead to the derangement of the hemostatic balance, which adds to the already-present hypercoagulable state related to the intense exercise [50]; at the same time, travel in cramped seating environments can favor venous stasis, decreased venous return, and hemoconcentration [47]. Previously, it has been already pointed out how long-distance travel by bus, car, train, or airplane triggers the mild activation of coagulation and an increase in calf volume, even among subjects with no pre-existing risk factors for VTE [50,51]. On top of that, diurnal rhythms (or circadian rhythms) also play a pivotal role in the hemostatic state of athletes, influencing various physiological processes, including sleep–wake cycles, hormone secretion, body temperature, and cardiovascular function [52]. Several studies have investigated the influence of diurnal rhythms on coagulation parameters, particularly in the context of sports and exercise [53,54,55]. Coagulation is one such process that can be affected by diurnal rhythms, inducing a hypercoagulatory and hypofibrinolytic state during the first hours of the morning, usually between 06:00 and 12:00 h [47,56,57]. It has been suggested that the timing of sporting events or training sessions may need to consider these diurnal variations to optimize performance and minimize the risk of adverse events. The identification of any risk factors and their correct management allows the athlete to be able to train and take part in competitions with greater safety. Therefore, it becomes essential to continuously monitor the state of health and, where necessary, proceed with in-depth tests that allow the timely identification of signs and symptoms of dangerous conditions.

### 3.2. Microtraumas and Sports

An increase in workload distributed non-adequately over time and/or unaccompanied by adequate athletic and muscular preparation can cause a sports injury. Traumas generally affect the musculoskeletal system, the bone segments of the joints, tendons, and muscles. Sports injuries can be of two types: acute and chronic, i.e., due to repeated microtraumas. Acute injuries are generally due to high-energy trauma, such as anterior cruciate ligament injuries that occur in football, whereas microtrauma injuries are typical of all the sporting activities characterized by a repeated gesture over time, such as tendinopathy of the swimmer’s rotator cuff [58] or the epicondylitis of the tennis elbow [59] or the golfer’s epitrochleitis [60]. The main causes of acute and chronic traumatic events are attributable to three types of dynamics, such as the following: conflict between athletes in sports with predominant physical contact (football 53%, wrestling 42%, and rugby 40%); accidental falls that can occur in sports that involve the use of mechanical and non-mechanical equipment (motorcycling 73% and cycling 59%); and twisting and strain of the limbs in individual sports where the aid of external agents is not required (volleyball 46% and gymnastics 37%) [61].

The damage caused by trauma can be of various degrees, such as a simple injury to the skin and muscles, without tissue lacerations, but also more serious effects; among these distortions are dislocations, fractures, and lesions [62]. Sports injuries can be muscular from strain, tear, or contusion; tendon lesions, such as insertional tendinopathies, tenosynovitis of the tendon sheaths, and tendinosis; and ligament and bone injuries, in particular stress fractures and real fractures. Furthermore, sports injuries can be typical of a specific sport; for example, injury to the anterior cruciate ligament of the footballer’s knee, injury to the rotator cuff of the swimmer’s shoulder, the tennis player’s epicondylitis, tendinitis of the patellar in the jumper’s knee, and tendonitis of the posterior tibialis in dancers.

Other sports that require repetitive gestures can instead cause injuries to the upper limbs, especially at the shoulder and elbow level, following trauma from falls or playing collisions (fractures and/or dislocations).

Amateur or professional athletes perform high-stress repetitive actions, applying significant force to anatomical structures. The result of this intense physical exercise is muscle hypertrophy that, in combination with position-dependent vessel occlusion and with repetitive movements, increases the risk of developing non-atherosclerotic peripheral arterial disease.

The treatment of sports injuries is represented by rest, ice, compression, and elevation of the limb (R.I.C.E), the first approach to always be implemented in a sports trauma while waiting for the diagnostic and interventional definition [63]. The advent of arthroscopy with minimally invasive techniques and the development of new biological and synthetic materials now makes it possible to carry out interventions to safeguard and repair residual joint components and/or biologically replace damaged components.

Arterial complications in athletes are often mislabeled as musculoskeletal injuries because both present similar symptoms. It is important to underline that an incorrect diagnosis and consequently an altered management of the athlete can delay recovery and cause serious consequences.

### 3.3. Doping and Thrombosis

The relationship between doping in sports and thrombotic events has been a topic of concern and research in the field of sports medicine. Throughout history, individuals have always strived to maximize their athletic abilities, seeking ways to boost physical performance, strength, and mental focus while minimizing fatigue and pain. This pursuit of excellence has undergone significant transformation in the past two centuries, driven primarily by substantial economic interests. Unlike the notion advocated by Baron de Coubertin that “the important thing in life is not the triumph but the struggle”, most athletes now prioritize achieving victory rather than solely focusing on the process. While the fatal consequences of doping are evident, it is crucial to recognize the wide range of detrimental effects it can have on the human body. Doping has been associated with various adverse outcomes spanning behavioral, skeletal, endocrine, metabolic, hemodynamic, and cardiovascular imbalances. Among the potential pathologies observed, cardiovascular complications such as heart ischemia (including acute coronary syndrome and acute myocardial infarction), cerebral ischemia (ranging from transient ischemic attacks to stroke), peripheral artery occlusive disease, and VTE are of particular concern. Despite these risks, professional athletes often underestimate the potential health hazards associated with doping, leading to its widespread use and, consequently, overuse. Doping refers to the use of prohibited substances or methods to enhance athletic performance. While the primary focus of doping control efforts is on improving fair competition and protecting athletes’ health, there is growing evidence suggesting a potential association between doping and an increased risk of thrombotic events. Thrombosis is the formation of blood clots within blood vessels, which can lead to serious medical conditions such as deep vein thrombosis (DVT) or pulmonary embolism (PE). Several factors related to doping may contribute to the development of thrombotic events in athletes: anabolic–androgenic steroids (AASs), erythropoiesis-stimulating agents (ESAs), growth hormone, and stimulants. AASs are a class of natural and synthetic testosterone derivates, heavily abused among athletes to enhance their performance, including gains in muscular mass and strength [64]. Their usage is predominant not only among professional athletes, but also in the general population (amateur athletes, trainers, and gym clients) [65]. AASs may cause a wide spectrum of clinical sequalae, which leads to an increased risk of arterial thrombosis. Side effects encompass hypertension, cardiomyopathy, stroke, pulmonary embolism, both fatal and nonfatal arrhythmias, and even myocardial infarction (MI), which can lead to sudden cardiac death [66]. The impact of anabolic–androgenic steroids (AASs) on coagulation is an area of concern, affecting various aspects of the coagulation system, potentially leading to an increased risk of thrombotic events. One potential effect of AASs on coagulation is an alteration in platelet aggregation. Studies have suggested that AAS use can increase platelet aggregation due to an increase in the platelet thromboxane A2, which may contribute to the formation of blood clots [66,67]. Additionally, AASs have been shown to affect plasma levels of certain coagulation factors, such as fibrinogen, factor VIII, and factor X, which play a role in the clotting process, with increased levels of homocysteine and diminished fibrinolysis functionality [66]. These changes can disrupt the delicate balance of the coagulation system and potentially increase the risk of thrombosis. ESAs, instead, are erythropoietin derivatives belonging to a group of medications acting as a stimulator of bone marrow erythropoiesis, clinically employed for the treatment of different forms of anemia resulting from chronic renal disease, malignancy, hematologic disorders, prematurity, and acquired immune deficiency syndrome [68]. For their ability to induce a rise in blood oxygen-carrying capacity that prompts maximal oxygen uptake during exercise, ESAs are exploited by some athletes to improve their physical performance, with unexplained death reported in the past due to a variety of cardiovascular complications including stroke, myocardial infarction, and VTE [66,68].

## 4. Blood Sample Analyses: Which Marker?

A sedentary lifestyle is considered an element capable of inducing major chronic diseases; strenuous physical activity can be viewed as a double-edged sword for coronary artery disease (CVD), endothelial function, immune function, and arterial thrombosis [69]. As already pointed out, the coagulation balance in athletes can depend on different elements, such as type of exercise, its intensity, duration, and training condition, leading to hemostatic distress. The body’s response to intense physical activities can resemble that which occurs in pathological states; this is largely due to the release of proinflammatory cytokines, particularly Interleukin-6 (IL-6), with plasma levels of the latter reported to be increased up to 50 times in marathon athletes soon after competitions [70]. Abundant cytokine production can lead to an inflammatory mechanism that can influence both procoagulant and anticoagulant pathways, interfering with platelet count, natural anticoagulant pathways, and fibrinolysis. While exercise-induced changes in coagulation parameters are generally well tolerated in healthy individuals, athletes with a history of clotting disorders or other medical conditions should consult their healthcare providers for personalized guidance. Additionally, monitoring coagulation parameters may be necessary for athletes undergoing specific medical treatments or interventions that could impact blood clotting. The main coagulation parameters include Prothrombin time (PT), activated partial thromboplastin time (APTT), fibrinogen, D-dimer, platelet count, and specific coagulation factors. PT measures the time necessary for blood plasma to clot and is an indicator of the extrinsic coagulation pathway, whereas aPTT evaluates the intrinsic coagulation pathway by measuring the time necessary for clot formation in the presence of specific activators. Coagulometry is the most common method for the determination of these two parameters. Fibrinogen is an essential component of the coagulation cascade as it acts as a substrate for the thrombin enzyme which gives life to fibrin monomers that by binding together will form an insoluble network, thanks to the intervention of coagulation factor XIII. One of the most common techniques for the evaluation of fibrinogen is the Clauss method, which involves the addition of thrombin to diluted plasma with measurement of the clot formation time, which will be inversely proportional to the fibrinogen concentration. D-dimer is a degradation product of fibrin, a protein responsible for the formation of thrombi in blood vessels. It represents a marker of hypercoaguability. D-dimer is measured by immunological techniques such as the Enzyme-Linked ImmunoSorbent Assay (ELISA) or turbidimetric methods. Rotational Thromboelastometry (ROTEM) and Thromboelastography (TEG) examine the viscosity of the blood to be assessed in real time as the clot forms and dissolves, offering a more complete picture than traditional tests. In fact, they allow the evaluation of the coagulation process over time, including the interaction between platelets, fibrinogen, and coagulation factors.

It has been reported that acute endurance exercise can lead to a hypercoagulable state due to increased plasma levels of Factor VIII (FVIII), thrombin–antithrombin III complex (TAT), prothrombin fragments 1 and 2 (F1 + F2), fibrinogen, and fibrinopeptide A, as well as a shorter aPTT [69,71]. A rise in tissue plasminogen activator (t-PA) with a reduction in plasminogen activator inhibitor-1 (PAI-1) levels are also reported following high-intensity short-duration exercise, suggesting that enhanced fibrinolysis can occur following extreme exertion [72]. As for FVIII, some studies reported how its activity can rise by 200–400%, depending on the magnitude of exercise [73]. Similar data were reported by Lippi et al. [74], who investigated the effect of middle-distance endurance exercise in middle-aged athletes on blood coagulation, showing a shortening of APTT clotting times with a remarkable increase in FVIII, von Willebrand antigen, and D-dimer values after the run, with D-dimer levels being a biomarker not only of the activation of coagulation but also of fibrinolysis. Particularly interesting in this study is the evaluation of the thrombin generation assay (TGA) performed on the citrated plasma of these runners. The TGA is a dynamic hemostasis test providing a global assessment of thrombin generation over time in a platelet pool plasma, offering information about inherited and acquired coagulopathies [75]. It was highlighted that the TGA registered a reduction in TGA-AUC (representing the area under the curve) and TGA-PK (representing the peak of thrombin generation) soon after the run. For the authors, this is suggestive of a state of hypercoagulability due to the exhaustion of thrombin recorded immediately after the run and it is linked to the neutralization of thrombin produced following the formation of thrombin–antithrombin complexes during endurance exercise [74]. Comparable findings were reported by Hanke et al. [76] using whole-blood coagulation with the help of Rotational Thromboelastometry (ROTEM) and platelet aggregation. This study highlights a reduction in clotting times, with an increased clot stability soon after a marathon, a triathlon, and immediately after long-distance cycling using a ROTEM test on citrated whole blood; on the other hand, the platelet aggregation test underlined platelet activation only during the marathon and on a reduced scale during the triathlon, but not during cycling. The authors concluded that running influences platelet activation, while physical activity triggers the activation of the coagulation system. Another study investigated the effects of acute and exhaustive exercise on the hemostatic and fibrinolytic responses in young adolescent males, recruiting 10 young, sedentary males subjected to an exhaustive stepping exercise [77]. The authors demonstrated that platelet count, aPTT, FVIII, von Willebrand Factor (vWF), and Tissue plasminogen activator (t-PA) were significantly elevated immediately after exercise, while PAI-1 was significantly decreased up to 1 h after exercise. During the first hour after exercise, all parameters return to baseline levels, except for FVIII and Plasminogen activator inhibitor-1 (PAI-1). 

These data obtained from the literature confirm the transient increase in the risk of cardiovascular complications during and soon after high-energy exercise, questioning the safety of strenuous activity.

## 5. Anticoagulant Therapy

Antithrombotic therapy in athletes suffering from VTE is not different from in those who do not practice any sport. However, particular attention is paid to sportspeople, given the potential bleeding risks they may face secondary to their activities [72]. A personalized balanced approach is auspicious in these groups of patients, given the need to consider both the thrombotic disease and the type of sport practiced. Historically, athletes necessitating antithrombotic therapy were temporarily or permanently forced to stop their sports activity due to possible onset of intracranial hemorrhage following a concussion [78]. Nowadays, guidelines indicate that athletes should be forbidden from high-impact physical activity when anticoagulated because of the bleeding risk [79], especially for sports with high concussion rates, like American football, ice hockey, soccer, basketball, and lacrosse. Generally, anticoagulation can last 3 months after provoked VTE occurs; thereafter, the athlete can return to their sporting activities. Unprovoked or recurrent VTE is a different story, requiring a lifelong therapy. The management of athletes requiring only a short-term anti-thrombotic therapy is not as complicated as for athletes with indications for long-term anticoagulation, who must face not only the risk of ending their sports career, but also psychosocial and financial consequences. To prevent these issues, in athletes that need lifelong anticoagulation, attempts have been made in order to embark on a short-term intermittent interruption of the therapy thanks to the favorable pharmacokinetic/pharmacodynamic (PK/PD) profiles of direct oral anticoagulants (DOACs), which typically have a short half-life [73], providing a more flexible, individualized therapy as opposed to vitamin K antagonist (VKA) anticoagulants. In the past, the use of VKA drugs did not allow this versatile approach, due to their prolonged anticoagulant action. The commercialization of anticoagulants with a faster mechanism of action, such as low-molecular-weight heparins (LMWHs) and DOACs, characterized by a “fast on/fast off” effect, allows an easier management of these patients when utilizing an intermittent anticoagulant approach [80,81]. For these reasons, due to management-related complications for sportspeople needing blood thinners, there is a need for a personalized therapy, considering not only the underlying disease and the type of sport practice, but also an individualized PK/PD study, allowing physicians to better understand how an athlete metabolizes the administered anticoagulant and what the level is at which there is still a minimal bleeding risk for the athlete.

## 6. Gut Microbiota and Thrombosis Modulator

The whole collection of bacteria, archaea, and eukarya colonizing the human gastrointestinal tract, named the gut microbiota, represents an interactive ecosystem that has co-evolved with the host, defining a mutually advantageous relationship; the latter is crucial for humans’ health establishment and maintenance, representing a subtle balance between health and disease conditions. Indeed, some of the most important roles carried out by the gut microbiota are to maintain the integrity of the mucosal barrier, to serve as a source of essential nutrients and vitamins, to protect against pathogens’ infiltration and colonization, and to properly fulfil immune functions [82]. Consequently, an imbalance of the intestinal microbiota, also known as gut dysbiosis, has been linked to the development and progression of several pathological conditions, including cardiovascular diseases (CVDs) [83]. Indeed, the systemic alterations occurring in the presence of a CVD can affect intestinal homeostasis since they can cause hypoxia and anaerobic cellular metabolism, resulting in pH lowering [84]. These factors promote a shift in the gut microbial community by increasing the abundance of enteropathogenic taxa, such as Candida, Salmonella, Shigella, and Campylobacter, and induce inflammation and increased gut barrier permeability [85]. In particular, since the gut microbiota acts as an endocrine organ through the secretion of bioactive molecules involved in the host’s health maintenance, recent research demonstrated that alterations in the microbial community, and consequently the release of toxic metabolites, are strictly related to cardiovascular pathology outbreak, given that they enter into the circulation and act as modifiers on the host [86,87].

In this context, trimethylamine (TMA) is produced from dietary phosphatidylcholine, choline, betaine, and L-carnitine by TMA lyase, a specific enzyme belonging to the gut bacteria; this bacterial metabolite is absorbed in the gut, reaches the liver via portal vein circulation, and is oxidized by host hepatic flavin monooxygenases (FMOs) to produce trimethylamine N-oxide (TMAO) [88]. It is noteworthy that, under physiological conditions, almost all of the produced TMAO is eliminated by the kidney or at the intestinal level [88]. However, in the presence of gut microbial dysbiosis, as observed in CVDs, TMA production increases and, consequently, TMAO circulating levels increase as well [88]. Since the initial discovery of TMAO, several clinical studies have been conducted to support the theory that increased TMAO plasma levels are directly proportional to the risk of a cardiovascular event [89]. Indeed, TMAO is involved in the modulation of cholesterol and sterol metabolism, cholesterol transport, and bile acid levels [90]. It is also known that TMAO chronically modulates the cardiovascular system, being involved in metabolic alterations, inflammation, ROS signaling, and cellular apoptosis as well; atherogenesis and cardiomyopathy are actually related to endothelial cells, vascular smooth muscle cells, cardiomyocytes, and inflammatory macrophages/foam cells, all targeted by TMAO [91]. In particular, the circulating levels of TMAO have been shown to be associated with CVD and to predict outcomes in the presence of multiple CVD phenotypes, including peripheral artery disease (PAD), coronary artery disease (CAD), acute coronary syndrome (ACS), and heart failure, besides the close association with thrombotic event risks, such as heart attack and stroke, that has been highlighted by numerous large-scale clinical cohorts [92].

Additionally, short chain fatty acids (SCFAs), even though linked to the host metabolism, are also produced in large quantities by the gut microbiota through anaerobic fermentation of dietary fibers which, escaping digestion by host enzymes in the upper gut, are then metabolized by the microbiota in the cecum and colon, representing the most abundant metabolites produced by the gut [93]. It has been demonstrated that SCFAs can directly activate G-coupled receptors, inhibit histone deacetylases, and serve as energy substrates, so they may affect various physiological processes and may contribute to health and disease [94]. The major products deriving from this microbial fermentative activity include acetate, propionate, and butyrate, which are absorbed into the portal blood and participate in various processes in the host, including lipid metabolism, glucose homeostasis, gut inflammation, and neurogenesis, but have been also linked to alterations in host blood pressure homeostasis, myocardial repair, and inflammation [95]. Moreover, recent studies provided further evidence that SCFAs are involved in other CVD processes, such as ischemia/reperfusion injury, cardiac repair following myocardial infarction, and impaired arterial compliance [92].

Another important metabolite produced by the gut microbiota is phenylacetylglutamine (PAGln); indeed, the dietary essential amino acid phenylalanine can be converted by the microbial porA gene into phenylacetic acid, which is further converted into PAGln and phenylacetylglycine in the liver. PAGln mediates cellular events through G-protein-coupled receptors, including α2A, α2B, and β2 adrenergic receptors, and represents a new CVD-promoting gut microbiota-dependent metabolite, since it was shown to enhance platelet responsiveness and to foster thrombosis potential [96].

In addition, the conversion of primary bile acids (BAs) to secondary BAs, which facilitate the absorption of dietary fats and fat-soluble molecules, is dependent on the gut microbiota as well. Secondary BAs are metabolized by the microbiota in the lower part of the small intestine and the colon and, entering the circulation, can act as hormones and regulate signaling pathways, including metabolism, inflammation, and energy expenditure [97]. In addition, BA secretion disorders can cause blood lipid abnormalities and have been implicated in the development of atherosclerosis and other cardiometabolic diseases [98].

Overall, diet is an important risk factor for CVD and the complex interactions between food and the gut microbiota and the related metabolites play a key role in cardiovascular health so much so that the use of probiotics, approved by the FAO and WHO, demonstrated their beneficial health impacts and is regarded as a potential therapeutic tool for CVD; therapies aimed at modulating the gut microbiota have been suggested as among the most promising strategies in this field [99]. In particular, supplementing the diet with prebiotics and/or probiotics that stimulate the expansion of specific microorganisms, such as Bifidobacterium and Lactobacillus, and resulting metabolites to improve metabolic, immune, and barrier function can be a useful integration for athletes. Indeed, the gut and the microbiota are both important organs for athletic performance because they are responsible for the delivery of water, nutrients, and hormones during exercise. Appropriate nutritional choices, for example avoiding fat and fiber, have been recommended to reduce the risk of gastrointestinal discomfort in elite athletes by ensuring rapid gastric emptying, water and nutrient absorption, and adequate perfusion of the splanchnic vasculature before competitions, with the aim to enhance healthy microbiota’s metabolites and to limit the toxic ones [100].

## 7. Conclusions

An adequate clinical evaluation and monitoring of laboratory parameters should be recommended to anyone who decides to start a program of physical activity, in particular to athletes and elite athletes. There is a widespread idea among physicians and coaches that athletes are not exposed to the risk of developing DTV, and furthermore the classical symptoms of VTE can be masked by sport-specific symptoms; because of this, early diagnosis and treatment are difficult. It is well described that some coagulation factors and platelet activity can exhibit diurnal variations. For example, levels of von Willebrand factor have been found to be higher in the morning compared to the evening. Similarly, platelet aggregation tends to be higher in the morning hours. In the context of sports, diurnal variations in coagulation may have implications for athletes, particularly regarding the risk of thrombosis and exercise-related injuries. In resting conditions, numerous markers of coagulation and fibrinolysis have shown diurnal rhythms; in particular, hypercoagulability, hypofibrinolysis, and increased blood viscosity occur between 06:00 and 12:00 h.

In this scenario, the realization of programs aimed at monitoring the health status of athletes could be crucial to avoid the occurrence, in some cases, of conditions that could threaten the athlete’s life.
